# omicplotR: visualizing omic datasets as compositions

**DOI:** 10.1186/s12859-019-3174-x

**Published:** 2019-11-15

**Authors:** Daniel J. Giguere, Jean M. Macklaim, Brandon Y. Lieng, Gregory B. Gloor

**Affiliations:** Department of Biochemistry, Schulich School of Medicine and Dentistry, Western University, London, N6A5C1 Canada

**Keywords:** Differential abundance, Data visualization, Compositional data, Effect plots, Exploratory data analysis, Differential expression

## Abstract

**Background:**

Differential abundance analysis is widely used with high-throughput sequencing data to compare gene abundance or expression between groups of samples. Many software packages exist for this purpose, but each uses a unique set of statistical assumptions to solve problems on a case-by-case basis. These software packages are typically difficult to use for researchers without command-line skills, and software that does offer a graphical user interface do not use a compositionally valid method.

**Results:**

omicplotR facilitates visual exploration of omic datasets for researchers with and without prior scripting knowledge. Reproducible visualizations include principal component analysis, hierarchical clustering, MA plots and effect plots. We demonstrate the functionality of omicplotR using a publicly available metatranscriptome dataset.

**Conclusions:**

omicplotR provides a graphical user interface to explore sequence count data using generalizable compositional methods, facilitating visualization for investigators without command-line experience.

## Background

High-throughput sequencing (HTS) technologies are commonly used to detect differential expression, where the expression of features (genes, operational taxonomic units, transcripts, etc...) in one group of samples is compared to another group. Microbiome, transcriptome and metagenomic studies share many characteristics, for example, in each case, a DNA (or cDNA) library is sequenced and reads are binned into features that represent a biological group in a given context [1]. Exploratory visualizations of the dataset enable identification of potential differences between conditions as well as outlier samples. Typical visualizations often include principal component analysis biplots [2], hierarchical clustering of features, and other plots to explore differential abundance [3].

Awareness of the compositional nature of HTS data has increased in the recent literature [4]. Briefly, the counts for sequencing reads obtained from an HTS experiment represent the relative proportions of reads in a sample, not the absolute abundance of DNA fragments. This is because the sequencing instrument itself imposes an arbitrary limit on the total number of reads collected (e.g., 25 million reads per run on an Illumina MiSeq), and therefore only collects data from a proportion of the molecules present.

There are currently no tools that provide a graphical user interface available to generate these visualizations using a compositional approach. Other graphical user interface tools have been proposed [5, 6], but do not offer compositional methods. Furthermore, many statistical models have been proposed to handle differential expression on a case-by-case basis, making it difficult to choose a statistical model that performs well on a given dataset. The compositional approach implemented for HTS data is generalizable, with minimal adjustable parameters needed for different experimental applications [1].

We have developed omicplotR as a graphical user interface for compositional read count tables in the R language. omicplotR incorporates ALDEx2 [7] to generate log-ratio transformed Bayesian posterior probabilities that can be used for differential abundance analysis. Since the compositional approach is generalizable, it can be applied to metagenomics [8], 16S rRNA gene sequencing [9, 10], metatranscriptomics [7, 11, 12], and any other relative count dataset. Here, we demonstrate usage with a vaginal metatranscriptome dataset [13] downloaded from the European Nucleotide Archive (project number PRJEB21446).

## Implementation

omicplotR is implemented using the Shiny framework in the R language, deployed on the Bioconductor repository [14]. The user interface launches in the user’s default browser, and the current version of omicplotR (1.4.3) has been tested on macOS version 10.14.5, Windows 7, and Ubuntu 18.04 with multiple browsers (Safaria for macOS, Chrome and Firefox for macOS 10.14.5, Windows 7 and Ubuntu 18.04). It is available for download from the Bioconductor repository (http://bioconductor.org). The development version is available at https://github.com/dgiguer/omicplotR. omicplotR currently requires R version > = 3.5.

## Getting started

**Fig. 1:**
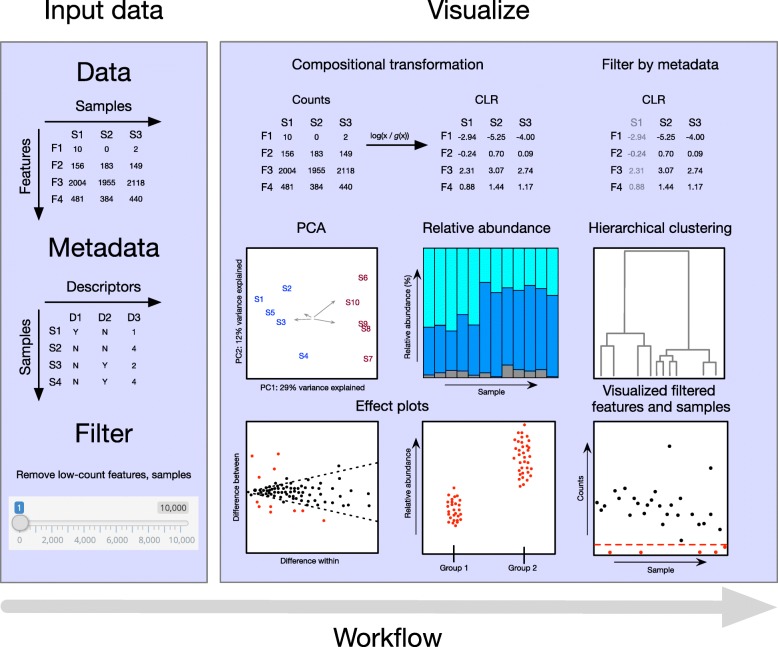
Workflow of omicplotR. The data input requires samples as columns, features by rows, whereas the metadata input descriptors as columns and samples by row. Count tables can be filtered to remove samples or features with low counts. After filtering, zero-imputation and a log-ratio transform is applied to the counts. A principal component analysis (PCA) biplot is typically the first exploratory visualization used. Several other plots are available to visualize differences between samples, features, and experimental conditions. Visualizing which features and samples have been removed by filtering is also possible. Plots are stylized representations of plots that can be generated by omicplotR

omicplotR launches from an R console with the command omicplotr.run(). The user-interface accepts a read count table as input, with the option to include a column of per feature taxonomic identifiers. Users can also input GO Slim annotated count tables that are obtained from the MGNify pipeline [15]. Optionally, metadata describing each sample can be included for filtering or visualizing sub-groups when plotting. Two example datasets are provided which can be accessed under the Example data tab, one from a vaginal microbiome [10] and another from a selective growth experiment [16]. The general workflow of omicplotR is shown in Fig. 1. The vignette provides a tutorial using the example datasets, and can be accessed by entering this command in the R console: browseVignettes(“omicplotR”).

## Data transformation

The count table can be transformed by zero-imputation [17] and a log ratio. This transformation is a compositionally appropriate alternative to many of the read-depth normalizations used by other differential analysis packages [8]. Optionally, pseudocounts can be used to quickly remove zeros for the log ratio [18].

## Visualizations

A useful first step in exploring sequencing count data is visualizing the data with a principal component analysis (PCA) biplot [2, 19]. This technique can be used to quickly estimate if there is a strong difference between experimental conditions. The default PCA biplot is not coloured, but can be coloured according to metadata categories. This allows the investigator to explore the differences within sub-groups to see which variable may explain the most variance. Both discrete and continuous variables are permitted as metadata, and the categorical frequencies are plotted as a histogram. The metadata can also be used to filter samples by group, allowing the user to explore sub-groups in the dataset. Removed features and samples can be visualized to examine how many are removed.

If a column of taxonomic identifiers was provided in the dataset (e.g., in 16S rRNA gene sequencing), the relative abundance of counts per feature can be displayed under the Relative abundance plots tab. Several options for hierarchical clustering and distance matrix methods are available by a drop-down menu.

Effect plots are used to visualize differentially abundant features by plotting the size of the difference between groups against the size of the difference within groups [3]. The interactive plots allow you to identify which features are differentially abundant and the uncertainty associated with their relative abundance. omicplotR also allows users to input pre-computed ALDEx2 tables for large datasets because the calculations can be time-consuming for large datasets.

For all visualizations, commented code to reproduce the plots can be downloaded with the filtering parameters chosen by the user, allowing the user to reproduce, report and adjust their visualizations.

## Results and discussion

To illustrate a use-case of omicplotR, we demonstrate its utility with a publicly available metatranscriptome dataset (European Nucleotide Archive project number: PRJEB21446). The GO slim annotation counts table was downloaded from MGNify [15], as well as the metadata from the paper [13]. The sample metadata was parsed into an acceptable format using code described in the Additional file 1.

The counts table and metadata can be imported into omicplotR by choosing “Select data” and “Select metadata” respectively. The investigator must check the box indicating the GO slim format. Viewing the data on this page by selecting “Check data” ensures that the data is in the correct format.

A coloured PCA biplot can be generated according to metadata (shown in Additional file 1: Figure S1). A histogram is generated to indicate the number of each variable present when metadata is present.

Exploring the data with exploratory PCA biplots shows a large separation on the first component, driven by the state of bacterial vaginosis (BV). This suggests that there is a strong and consistent difference between samples positive and negative for BV, and warrants further investigation. If there was no strong separation between experimental conditions when coloured by experimental condition, it would likely indicate no real difference between the experimental conditions. Using the PCA biplot function in omicplotR is a quick way to determine whether a difference exists between conditions, and can provide an estimate of how strong the difference(s) may be.

Lastly, effect size plots were generated (Additional file 1: Figure S2). omicplotR incorporates the ALDEx2 R package, and allows investigators to compare groups for differential abundance (expression, usage, etc...). In this case, an effect plot was generated to compare samples that were positive or negative for bacterial vaginosis. Investigators can interact with this plot by hovering their mouse over a point to visualize the distribution of effect sizes for a given feature. This allows for quickly interpreting the difference within groups, as well as the difference between groups, useful for evaluating borderline cases of differential expression. Differences driven by outliers can be easily detected with this visualization. For example, the GO:0051538 number corresponds to a reduction of the “iron-sulfur cluster binding” function in the BV negative group. In future releases, we will add statistical tests such as analysis of similarities and permutational analysis of variance [20] for testing the difference between groups in a statistical manner.

In the development version, we implemented an option to download datasets from the EBI MGnify database using an accession number, allowing the user to easily import publicly available datasets. More visualizations are available in omicplotR depending on the dataset, and use-cases are described in detail in the vignette. For example, omicplotR allows visualization of the relative abundance of taxa and hierarchical clustering for 16S rRNA gene sequencing datasets.

Using data downloaded directly from MGNify, the investigator can visualize the relative abundance of features by GO Slim annotation. The functions are separated into biological categories, and are plotted as a stripchart. Similar to the PCA biplots, each sample can be coloured according to metadata to explore the differences between groups, and experimental conditions.

## Conclusions

There is growing awareness of the compositional nature of high-throughput sequencing data. However, there is currently no graphical-user interface available that enables this approach for researchers without scripting experience. omicplotR is available as a Bioconductor package to facilitate analysis and exploration of omic data using compositionally appropriate methods. No scripting knowledge is required to use this tool. Reproducibility and fine-tuning of visualizations is achieved using commented downloadable scripts. omicplotR is capable of visualizations typically used in exploratory pipelines for differential abundance analysis. The generalizability of the compositional approach allows this tool to be applied to 16S rRNA gene sequencing, metagenomics, metatranscriptomics, and most other relative datasets in the format of count data.

## Supplementary information

**Additional file 1: Figure S1**. Coloured principal components analysis (PCA) biplot. **Figure S2.** Interactive effect plot.

## Data Availability

The dataset analyzed during the current study is available from the European Nucleotide Archive Study PRJEB21446 [13] at https://www.ebi.ac.uk/ena/browser/view/PRJEB21446, with download instructions in the supplemental material. The omicplotR source code is available at https://github.com/dgiguer/omicplotR (DOI: 10.5281/zenodo.3470142). Project name: omicplotR. Project home page: https://bioconductor.org/packages/devel/bioc/html/omicplotR.html, https://github.com/dgiguer/omicplotR. The version described in this manuscript is the development version. Operating systems: Platform independent. Programming language: R. Licence: MIT. Any restrictions to use by non-academics: None.

## Abbreviations

BV: Bacterial Vaginosis
HTS: High-throughput sequencing
PCA: Principal component analysis
