# Supplementary material for: omicplotR: visualizing omic datasets as compositions
Source: BMC Bioinformatics. 2019 Nov 15;20:580. doi: 10.1186/s12859-019-3174-x (PMC6858670; doi:10.1186/s12859-019-3174-x)
Supplement: Supplementary file 1 — Additional file 1: Figure S1. Coloured principal components analysis (PCA) biplot. Figure S2. Interactive effect plot. [file 12859_2019_3174_MOESM1_ESM.pdf]

Supplemental information

Daniel Giguere
Jean Macklaim
Brandon Lieng
Greg Gloor

03 October 2019

Extracting sample metadata from paper

The following code was used to extract metadata from the excel worksheet provided as metadata in the original paper (DOI: 10.1128/mSphereDirect.00262-18).

```
R
library(readxl)

# supplement downloaded as "inline-supplementary-material-1.xlsx" the
# above paper
# A1:R41 specifies only certain ranges, i.e., excludes last couple of
# non-metadata lines present in sheet 1
df <- read_excel("inline-supplementary-material-1.xlsx", sheet = 1,
range = "A1:R41")

# rewrite the first sheet of the supplementary as a tab-separated table
write.table(df, file = "metadata.txt", sep = "\t", row.names = FALSE,
col.names=TRUE, quote = FALSE, na="NA")

# quit R
q()
n

# this requires unix based system.
# adds sample accession numbers to match with GOSlim counts table.
# From EBI Study: PRJEB21446, click select columns, and select ONLY study
# accession, experiment accession, sample accession, run accession (this is
# what the count table uses), read count, sample alias (how we will match the
# GOSlim file). There should be 6 columns, with the Run accession as the 4th.
# both files are in the same order so adding by index works
# in first file, store column 4 of line in a
# print a, a tab, and the entire line from the metadata.txt file to a new file
awk 'NR==FNR{a[NR]=$4;next}{print a[FNR]"\t"$0}'
EBI_metadata.txt metadata.txt > final_metadata.txt

# the file "final_metadata.txt" is now ready to be imported into `omicplotR`
```

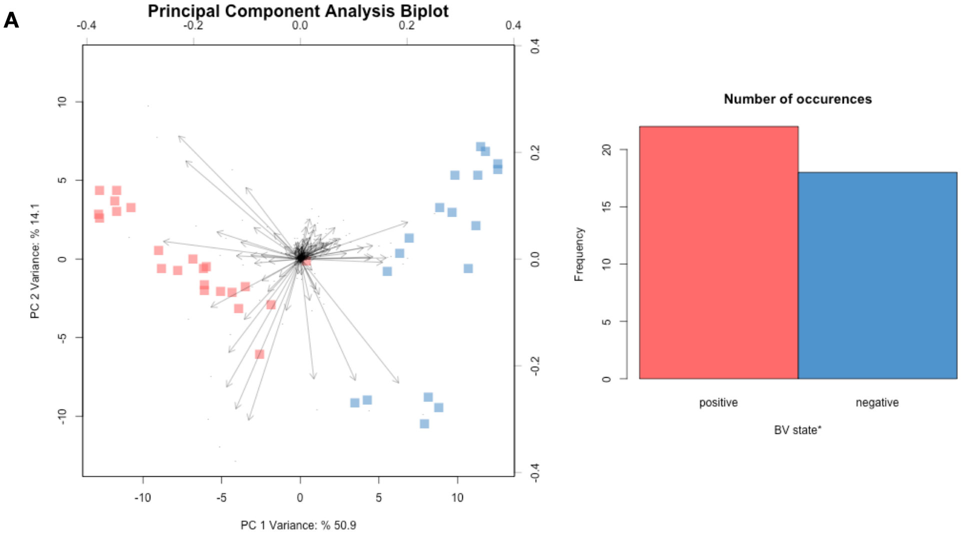

Figure 1: Coloured principal components analysis (PCA) biplot. Metadata is extracted from the input, and used to colour the samples by condition. In this case, samples are coloured according to whether or not they are positive for bacterial vaginosis (BV). Plots were generated directly from omicplotR and saved as .png files.

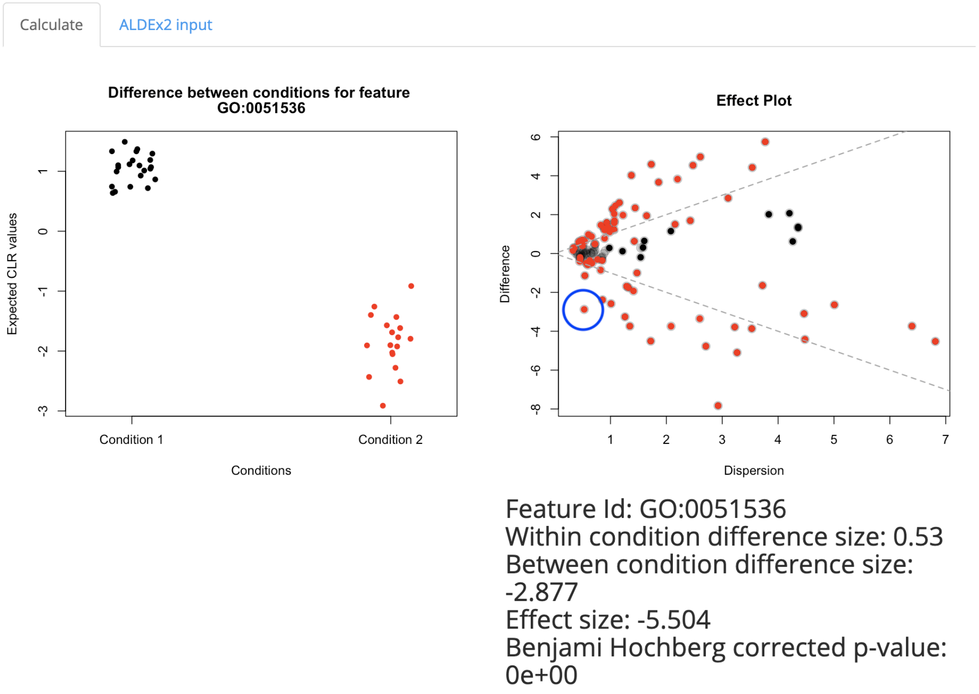

Figure 2: Interactive effect plot. Conditions tested were from metadata, using ALDEx2 to compare samples that were positive or negative for bacterial vaginosis (BV state in metadata). The figure on left was reactively generated by hovering the pointer over the point on the effect plot figure. Information about point such as difference within groups, difference between groups, and effect size are also reactive. The point circled in blue was the one that was being visualized. This figure was generated directly using omicplotR.
